# Clinical Application of Blood Biomarkers in Neurodegenerative Diseases—Present and Future Perspectives

**DOI:** 10.3390/ijms25158132

**Published:** 2024-07-25

**Authors:** Daria Krawczuk, Agnieszka Kulczyńska-Przybik, Barbara Mroczko

**Affiliations:** 1 Department of Neurodegeneration Diagnostics, Medical University of Białystok, 15-089 Białystok, Poland; daria.krawczuk@sd.umb.edu.pl (D.K.); agnieszka.kulczynska-przybik@umb.edu.pl (A.K.-P.); 2 Department of Biochemical Diagnostics, Medical University of Białystok, 15-089 Białystok, Poland

**Keywords:** neurodegeneration, Alzheimer’s disease, Parkinson’s disease, multiple sclerosis, Creutzfeldt–Jakob disease, blood biomarkers

## Abstract

Neurodegenerative diseases are a group of complex diseases characterized by a progressive loss of neurons and degeneration in different areas of the nervous system. They share similar mechanisms, such as neuroinflammation, oxidative stress, and mitochondrial injury, resulting in neuronal loss. One of the biggest challenges in diagnosing neurodegenerative diseases is their heterogeneity. Clinical symptoms are usually present in the advanced stages of the disease, thus it is essential to find optimal biomarkers that would allow early diagnosis. Due to the development of ultrasensitive methods analyzing proteins in other fluids, such as blood, huge progress has been made in the field of biomarkers for neurodegenerative diseases. The application of protein biomarker measurement has significantly influenced not only diagnosis but also prognosis, differentiation, and the development of new therapies, as it enables the recognition of early stages of disease in individuals with preclinical stages or with mild symptoms. Additionally, the introduction of biochemical markers into routine clinical practice may improve diagnosis and allow for a stratification group of people with higher risk, as well as an extension of well-being since a treatment could be started early. In this review, we focus on blood biomarkers, which could be potentially useful in the daily medical practice of selected neurodegenerative diseases.

## 1. Introduction

Neurodegeneration, in general, refers to direct cell death by necrosis or the delayed process of apoptosis [1]. The main hallmarks of neurodegeneration are neuronal cell death, inflammation, pathological protein aggregation, cytoskeletal abnormalities, altered mitochondrial function, aberrant proteostasis, and DNA and RNA defects [2].

Neurodegenerative diseases (NDs) can be classified according to major clinical features, anatomical distribution of pathological changes, or major molecular abnormalities [3]. Known risk factors for developing neurodegenerative disease are mainly age and genetic factors but also gender, low level of education, metabolic condition, oxidative stress, inflammation, head injuries, environmental factors, diabetes, infections, etc. [4].

One of the most common features of neurodegenerative diseases is neuroinflammation. Chronic neuroinflammation results in excessive pro-inflammatory cytokine and reactive oxygen species (ROS) excretion by microglia and astrocytes. This leads to alteration of synapses, impaired neurogenesis, and death of neuronal cells [5].

The diagnostic process of neurodegenerative diseases is challenging. Especially due to the lack of specific symptoms at the earliest stages of the disease, or the atypical variants of symptoms that may overlap, it is difficult to differentiate them. Neuropathological analysis of brain tissue is considered the gold standard; however, it cannot be included in the process of clinical diagnosis [6]. The diagnostic process is mainly based on clinical assessment. Functional imaging is commonly used for the exclusion of alternate diagnosis but a growing body of research points to the fact that imaging could have greater specificity for neurodegenerative diseases and, thus, may serve as a tool in the early diagnosis of dementia and parkinsonism [7].

**Figure 1:**
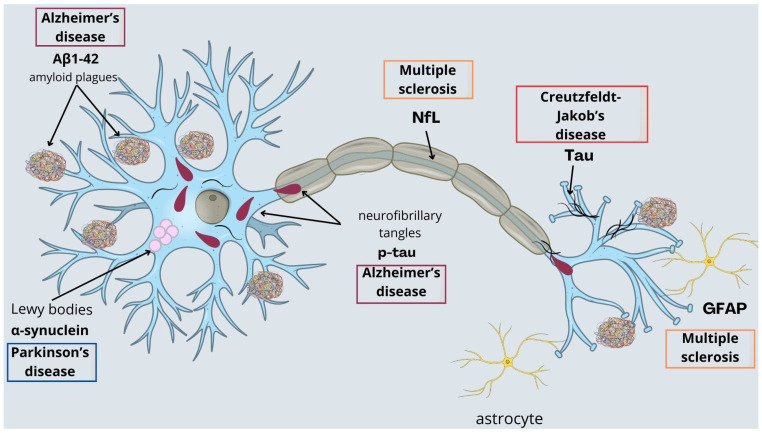
Protein biomarkers reflecting pathological processes in neurodegenerative diseases.

In recent years, huge progress has been made in the field of protein biomarkers for neurodegenerative diseases. Due to the fact that biomarkers reflect biological and pathological processes ongoing in the nervous system, they may also play a crucial role in understanding the pathogenesis of neurodegenerative diseases (Figure 1). Protein biomarkers could be valuable tools not only for the diagnosis of NDs but also for predicting future cognitive decline in healthy individuals and the monitoring of progression to dementia among cognitively impaired patients. Moreover, some markers may be used in the assessment of treatment efficacy, enabling the development of new therapeutic strategies [8]. Cerebrospinal fluid (CSF) is an optimal source of biomarkers as it is in direct contact with the extracellular space of the brain and it reflects biochemical changes that are taking place. However, the procedure of collecting CSF (lumbar puncture) is highly invasive and burdened with side effects (e.g., nausea, headache, backache, fatigue). Thus, there is a growing need for other, more approachable diagnostic material, such as blood, saliva, or other fluids. Blood-based biomarkers are beneficial due to minimal invasiveness with blood collection, simple procedures, and low costs. For decades different analytical platforms were used to measure plasma biomarkers. One of the most common techniques is the enzyme-linked immunosorbent assay (ELISA), which was initially developed for CSF assay. However, in terms of plasma, it turned out to have methodological issues that significantly affected the performance of the ELISA. Multiple Analyte Profiling (xMAP technology) was one of the first technologies used as an alternative to ELISA. As it simultaneously measures many analytes, it reduces the amount of the sample and improves the general workflow for biomarker analyses. Electrochemiluminescence (ECL) and Mesoscale Discovery (MSD) are increasingly moving forward as surrogates of traditional ELISA and xMAP technologies as both platforms showed improved sensitivities to detect lower levels of biomarkers. The immunoprecipitation and mass spectrometry (IP-MS) methods are also reliable for protein quantification and have shown robust results for Aβ and pTau in plasma. To date, findings from head-to-head studies indicate that MS-based methods are the most accurate and reliable to analyze plasma biomarkers, although further validation studies are necessary [9]. However, more prospective studies are necessary to determine their diagnostic properties. The recently developed SIMOA (single molecular array) technology offers improved sensitivity and enables measurements of very small amounts with high diagnostic accuracy. SIMOA technology relies on single-molecule arrays and the simultaneous counting of singulated capture microbeads. From a clinical point of view, the most reliable technique for the quantitative assessment of blood biomarkers seems to be fully automated methods, such as the SIMOA or ECL [9]. 

The aim of this review was to summarize current knowledge about the utility of the most promising blood biomarkers in the clinical practice of common neurodegenerative diseases, such as Alzheimer’s disease, Parkinson’s disease, multiple sclerosis, and Creutzfeldt–Jakob disease.

## 2. Alzheimer’s Disease

Alzheimer’s disease (AD) is a complex, progressive, neurodegenerative disease that is characterized by a decline in cognition. It is the most common type of dementia, accounting for 60% to 80% of all cases [10]. Approximately forty-four million people worldwide have dementia and this number could triple by 2050 due to an aging society [11]. Thus, AD is categorized by the World Health Organization as a disease of public health priority [12]. It is suggested that about 20% of women and 10% of men will eventually develop AD [13]. Estimated survival time (in Europe) after diagnosis of AD is 6 years. This number varies according to the stage of the disease [14].

Alzheimer’s disease has a long asymptomatic period which may last even 20 years. During this time, neurodegenerative processes in the brain connected with the loss of cholinergic neurons are taking place. After an 80% loss of those neurons, cognitive reserve is crossed and disturbing symptoms start to occur [15].

The neuropathological changes typical of AD include brain atrophy in the hippocampus and cortex as a result of the loss of neurons in these areas. The formation of amyloid deposition is particularly evident in the pre-olfactory cortex, entorhinal cortex, the CA1 region of the hippocampus, and the cortical associative regions [16]. Neurofibrillary tangles (NFTs), on the other hand, are accumulated mainly in the brainstem nuclei, especially the substantia nigra, and locus coeruleus in some tauopathies [17].

There are three stages of AD: preclinical AD, mild cognitive impairment (MCI) (due to AD), and dementia [18]. During the MCI stage, patients may exhibit cognitive decline, such as loss of memory; however, it does not affect their everyday life. Approximately 16% of sixty-five-and-older people have been diagnosed with MCI. Additionally, 15% of MCI patients will develop dementia in 2 years [19]. Two types of MCI may be distinguished: amnestic, with symptoms connected to memory loss, and non-amnestic, with impaired language, executive, and visuo-spatial function [20]. Preclinical AD and MCI are crucial stages due to the fact that biomarkers reflecting pathological processes typical for AD have already increased. Blood-based biomarkers allow early identification of changes in protein levels and, thus, faster decisions about lumbar puncture [21].

As reported by the National Institute on Aging and Alzheimer’s Association workgroup, proper diagnosis can be based on clinical and pathophysiological conditions, as well as assessment of CSF biomarkers and neuropsychological tests. Nonetheless, a definite diagnosis is only available via autopsy [21]. 

In 2018, the National Institute on Aging-Alzheimer’s Association (NIA-AA) reported that the highest probability of identifying AD pathology is achieved by combining markers of Aβ (A) with p-Tau (T) pathology, which could be assessed in CSF [21]. However, the literature data provide evidence that also blood biomarkers could be valuable diagnostic tools (Table 1).

### 2.1. Biomarkers of Amyloid Pathology

According to the amyloid-cascade hypothesis, the main cause of Alzheimer’s disease is the intra- and extra-neuronal accumulation of misfolded Aβ, generating toxic oligomeric species. Amyloid beta (Aβ) is a product of amyloid precursor protein (APP) proteolysis by BACE1 (beta-site APP cleavage enzyme 1) and γ-secretase complex. APP cleavage is completed by a non-amyloidogenic or amyloidogenic pathway. The first one takes place continuously in physiological conditions whereas non-amyloidogenic only takes place in pathological conditions. In the non-amyloidogenic pathway, APP is cleaved by α-secretase releasing sAPPα and C83 bound to the membrane, which, as well, is cleaved by γ-secretase. This cleavage, however, results in the release of p3 fragments and the amyloid precursor protein intracellular domain (AICD) without pathologic properties [40]. In the amyloidogenic pathway, APP is cleaved by β-secretase, resulting in the release of soluble APP β (sAPPβ) and C99, which is bound to the membrane. C99 is then cleaved by γ-secretase to release Aβ and the AICD. The accumulation of Aβ peptides in extracellular medium outside neurons leads to their aggregation and forming amyloid plaques. The Aβ peptide usually contains 37–43 amino acids, depending on the cleavage of γ-secretase, with Aβ1-40 being the most common isoform and Aβ1-42 being the most toxic. Isoforms with 42 and 43 amino acids are more prone to aggregate, forming oligomers and fibrils [41]. Progressive deposition of Aβ1-42 plaques results in neuronal damage and synaptic dysfunction, which leads to neurodegeneration [42]. 

It was postulated that measurements of Aβ species in the blood may significantly improve diagnostic accuracy of the clinical diagnosis of AD due to their pivotal role in AD pathogenesis. Moreover, it was postulated that the Aβ1-42/1-40 ratio allows for risk prediction of the risk for developing AD [43]. However, cross-sectional studies examining the plasma concentration of Aβ1-42 in AD patients have produced inconsistent results. Such difficulties may be caused by poor analytical performance of commonly used techniques. Variations between studies may come from many pre-analytical components of the assay, such as the choice of anticoagulant, needle size, order of blood draw, whether the sample undergoes a denaturation/extraction step or the addition of protease inhibitor, storage conditions, the use of plasma or serum, and the number of freeze/thaw cycles [44]. Moreover, as the concentration of Aβ1-42 in blood is especially low, more precise techniques are needed. 

Plasma levels of Aβ1-42 and Aβ1-40 are decreased in AD whereas the plasma Aβ1-42/Aβ1-40 ratio is already decreased in the MCI stage and even more in AD compared to controls [22]. Similar findings were observed by other researchers [23,24,25]. Conversely, Hansson et al. examined the diagnostic usefulness of plasma Aβ1-42 and Aβ1-42/Aβ1-40 ratio in predicting conversion to AD among MCI patients and found that neither of the baseline concentrations of Aβ1-42, nor the Aβ1-42/Aβ1-40 ratio, could predict conversion of MCI to AD compared to CSF analysis. It was explained by the lack of correlation between CSF Aβ1-42 and blood, thus indicating that in terms of incipient AD, CSF analysis is still more reliable than blood [45]. 

A growing body of research focuses on the use of the Aβ1-42/Aβ1-40 ratio as a prescreening tool, especially to limit the number of positron emission tomography (PET) examinations. Recently, a group of researchers showed that plasma Aβ1-42/Aβ1-40 ratio detected changes in CSF Aβ levels and/or amyloid-PET status in the continuum of AD. Moreover, the plasma Aβ1-42/Aβ1-40 ratio correlated similarly with amyloid-PET for both platforms. Thus, the use of the plasma Aβ1-42/Aβ1-40 ratio may serve as a non-invasive prescreening tool and reduce the number of necessary PET scans [26]. Rabe et al. evaluated the clinical performance of the plasma Aβ1-42/Aβ1-40 ratio for amyloid positivity prescreening. It was shown that the plasma Aβ1-42/Aβ1-40 ratio could potentially rule out amyloid pathology in populations with low-to-moderate amyloid positivity prevalence; however, this ratio was characterized by lower concordance as compared to the CSF ratio. Thus, the use of the plasma Aβ1-42/Aβ1-40 ratio as a prescreener should be carefully evaluated [46]. A similar study demonstrated that the plasma Aβ1-42/Aβ1-40 ratio has the potential as a prescreening tool to identify the earliest AD changes in cognitively normal individuals with subjective cognitive decline (SCD) [27]. Plasma ratio positively correlated with CSF Aβ1-42 levels and presented high diagnostic performance as compared to measurements of Aβ1-42 alone (AUC = 0.77 for ratio and 0.66 for Aβ1-42). Moreover, combining this ratio with age and APOE ε4 status resulted in an accuracy above 80%. Subjects with abnormal amyloid-PET status showed lower levels of plasma Aβ1-42 compared to normal amyloid-PET. The same tendency was observed for the Aβ1-42/Aβ1-40 ratio. The authors also revealed an association between a lower Aβ1-42/Aβ1-40 ratio and increased risk (two-fold) of clinical progression to MCI or dementia among patients with subjective cognitive decline. As for the predictive value, the Aβ1-42/Aβ1-40 ratio was lower in SCD subjects who progressed to MCI or dementia compared to those who remained stable [27].

It should be noted though that the plasma Aβ1-42/Aβ1-40 ratio is decreased only by 10–20% in subjects with Aβ pathology, compared to 40–60% for the CSF Aβ1-42/Aβ1-40 ratio. A possible explanation is that plasma Aβ is affected by Aβ metabolism outside the brain. Moreover, the CSF Aβ1-42/Aβ1-40 ratio is less prone to variations in its optimal cut-point. Combining the plasma Aβ1-42/Aβ1-40 ratio with other biomarkers may increase the diagnostic value and remove possible pre-analytical factors [47].

### 2.2. Biomarkers of Tau Pathology

Tau is a soluble protein that occurs in six main isoforms in the human central nervous system (CNS) [48]. In physiological conditions, tau provides stabilization and facilitates axonal transport by binding to microtubules [49]. In Alzheimer’s disease, however, tau undergoes hyperphosphorylation, leading to dissociation from microtubules and aggregation into paired helical filaments (PHFs) present in NFTs [50]. NFTs emerge from the entorhinal cortex and disseminate in the limbic and associated areas, reaching the hippocampus and neocortex [51]. Such deposition affects synaptic transmission, axonal transport, and signal transduction and the cell gradually undergoes degeneration [52].

Studies by Mattson et al. revealed that plasma pTau-181 and pTau-217 are associated with both Aβ-plaques (early stage) and NFTs (later stage) [53]. According to research by Simren et al., pTau-181 was the only one among the Aβ1-42, Aβ1-42/Aβ1-40 ratio, tau, NfL, and GFAP that provided high diagnostic accuracy to distinguish AD dementia from cognitively unimpaired individuals (AUC = 0.91) [28]. In agreement with that is also another study presenting the diagnostic accuracy of plasma pTau-181. Karikari et al. demonstrated that plasma pTau-181 distinguished AD from frontotemporal dementia (FTD), young adults, cognitively unimpaired older adults, and MCI. Moreover, authors found that plasma pTau-181 distinguished Aβ-positive cognitively unimpaired older adults from Aβ-negative cognitively unimpaired older adults and young adults. Plasma pTau-181 was increased in AD compared to several Aβ-negative neurodegenerative disorders. In the context of differential diagnosis, plasma pTau-181 differentiated AD from vascular dementia, progressive supranuclear palsy or corticobasal syndrome, and Parkinson’s disease or multiple system atrophy. Plasma pTau-181 predicted tau PET positivity (AUC and accuracy >90%) and amyloid-PET positivity (AUC = 0.88 and accuracy > 80%) in patients with MCI, AD, and FTD. Furthermore, plasma pTau-81 was a better predictor of AD than age, APOE ε4 genotype, or even both of them combined. Plasma pTau-181 more accurately predicted AD than plasma Aβ1-42, Aβ1-42/Aβ1-40 ratio, total tau, or total tau/Aβ1-42 ratio. According to these findings, blood pTau-181 is a useful test for supporting AD diagnosis, especially at the earliest stages [54].

It was established that also other isoforms of pTau could be valuable diagnostic tools. Both plasma pTau-217 and pTau-181 concentrations were increased in clinical AD compared to cognitively normal controls. Additionally, pTau-217 presented slightly better diagnostic performance than pTau-181 in the differentiation of clinical AD and frontotemporal lobar degeneration (FTLD) (AUC = 0.93 vs. 0.91). Moreover, pTau-217 was a stronger indicator of amyloid-PET positivity than pTau-181. Those findings suggest that both isoforms of pTau have an excellent diagnostic performance in differentiating AD patients from other neurodegenerative diseases **[29]**. Similarly, plasma pTau-217 assay differentiated AD and controls with high diagnostic accuracy (AUC = 0.98) and pTau217 levels were 3.9-fold higher in individuals with AD [55].

In the cross-sectional study conducted by Palmqvist et al., it was reported that plasma pTau-217 discriminated AD from other neurodegenerative diseases (PD, PSP, VaD, bvFTD, MSA) and distinguished patients with neuropathologically defined AD from those without AD pathology. The diagnostic accuracy of pTau-217 was notably higher than plasma pTau-181, neurofilament light chain, and MRI measures, but not significantly different compared with CSF pTau-217, pTau-181, and tau-PET. Additionally, pTau-217 levels correlated with cerebral tau tangles and discriminated normal vs. abnormal tau-PET scans with higher accuracy than plasma pTau-181, plasma NfL, CSF pTau-181, the CSF Aβ1-42/Aβ1-40 ratio, and MRI measures. Plasma pTau-217 levels were also higher among PSEN1 mutation carriers compared to noncarriers [56]. Plasma pTau-181 and pTau-217 were significantly higher in the MCI and dementia group compared to cognitively unimpaired patients. Moreover, both pTau measures were excellent predictors of abnormal amyloid-PET and tau PET [57]. Barthelemy et al. investigated the role of the ratio of pTau-217 to the non-phosphorylated tau (%pTau-217). The authors suggested that the ratio is less affected by confounding factors. It was revealed that blood plasma %pTau-217 exhibited great diagnostic performance (AUC 0.94) in distinguishing individuals with and without symptomatic AD. Moreover, the performance of %pTau-217 was clinically equivalent to the CSF pTau-181/Aβ1-42 ratio and CSF Aβ1-42/1-40 ratio. Furthermore, among MCI and mild dementia patients, plasma %pTau-217 classified Aβ PET status with an accuracy of approximately 90% [58]. 

In a follow-up study, plasma p-Tau217 was measured repeatedly for up to 6 years in cognitively unimpaired and MCI patients. MCI patients who converted to AD had increased pTau-217 levels compared to those who did not. Moreover, longitudinal growth in pTau-217 concentration correlated with worsening cognition and brain atrophy.

Those findings indicate that this protein increases during the early stages of Alzheimer’s disease and may be useful in monitoring disease progression [33]. Ashton et al. investigated the utility of plasma pTau-231. It turned out that plasma pTau-231 differentiated AD patients from amyloid-β negative cognitively unimpaired adults with high diagnostic accuracy. Furthermore, it distinguished AD patients from those with non-AD neurodegenerative disorders and from amyloid-β negative MCI patients with similar accuracy [34]. Based on the aforementioned findings, pTau-181, pTau-217, and pTau-231 demonstrate great performance, especially during the preclinical stages of Alzheimer’s disease. 

Another tau isoform recently discovered is pTau-212. In a study conducted by Kac et al., plasma pTau-212 levels were significantly higher in the AD-MCI and AD dementia groups compared to non-AD MCI and SCD groups. Similar results were obtained for p-Tau217. The diagnostic accuracy of plasma p-Tau212 was similar to p-Tau217 but higher than p-Tau181 and p-Tau231. Furthermore, plasma pTau-212 and pTau-217 showed similar performances to CSF, thus showing its high potential for application in clinical diagnosis, population screening, and monitoring patients eligible for anti-AD therapies [30].

### 2.3. Biomarkers of Neurodegeneration and Synaptic Dysfunction

#### 2.3.1. NfL

One of the well-known biomarkers of neurodegeneration is the NfL. Neurofilaments (Nfs) are major intermediate filament proteins that constitute filaments of neurons. Together with the other four neuronal intermediate filament proteins, namely, neurofilament heavy chain, neurofilament medium chain, alpha-internexin, and peripherin, the NfL assembles into neurofilaments, which are important for dendritic branching and growth and stability of axons in both central and peripheral nerves and for post-traumatic axonal regeneration [31]. NfLs are released to the CSF and blood after axonal damage and neurodegeneration in significant amounts [32]. CSF and blood levels of the NfL increase relatively to the degree of axonal damage, suggesting that its levels may have a prognostic value in a wide range of neurological disorders. Recently developed assays enable NfL quantification in blood, which makes it a useful tool, especially in monitoring disease progression, when multiple assays are needed [59].

The plasma NfL level was significantly higher in the MCI and AD groups compared to non-demented controls. The authors established a cut-off value of 25.7 pg/mL with sensitivity, specificity, and accuracy of 84%, 78%, and 82%, respectively [60]. In another study, a group of researchers observed that the plasma concentration of the NfL was higher in MCI and AD patients compared to healthy controls. Moreover, plasma NfL levels increased with time in the MCI group (2.7 ng/L per year) and AD group (4.9 ng/L per year). In all groups, higher plasma concentrations of the NfL were associated with accelerated reduction in FDG-PET measures, expansion of ventricular volume, and lower MMSE scores. Additionally, a greater increase in NfL level was in line with accelerated elevation in tau and pTau levels and white matter lesions among MCI patients. Those findings suggest that the plasma NfL is correlated with a course of AD and may serve as a part of the AD biomarkers panel [37].

It should be noted, however, that increased NfL concentration is not specific for AD but, also, other neurodegenerative diseases are characterized by increased levels of this protein. Thus, instead of the diagnostic process, it may be more useful for monitoring AD progression (rate of cognitive decline).

#### 2.3.2. GFAP

GFAP (glial fibrillary acid protein) is also a promising candidate. GFAP is a monomeric filament protein almost exclusively expressed by mature astrocytes. Due to hyperplasia of the astrocytes population in the CNS, it is elevated in the CSF and blood [61,62].

An interesting study performed by Bendet et al. showed that plasma GFAP levels were significantly higher among individuals with preclinical AD with the highest values at the symptomatic stages of the disease. Surprisingly, plasma GFAP discriminated Aβ-positive cognitively unimpaired individuals from Aβ-negative cognitively unimpaired individuals more accurately than CSF GFAP concentrations [38]. Chatterjee et al. investigated whether increased plasma GFAP concentrations preceded the onset of the clinical symptoms of AD in cognitively normal older adults at risk of AD. The results showed significantly higher plasma GFAP concentrations in the Aβ+ group (high brain Aβ load) compared to Aβ− subjects (low brain Aβ load). Moreover, the plasma GFAP was also higher in the Aβ+ SMCs (subjective memory complaints) group compared to Aβ− SMCs. Interestingly, similar results were reported in non-SMC patients whose plasma GFAP was significantly higher in Aβ+ non-SMCs compared to Aβ− non-SMCs. Those findings suggest that plasma GFAP levels are increased in cognitively normal older adults with high brain Aβ load, meaning that it may serve as an early blood-based biomarker to identify people at risk of AD before the onset of clinical symptoms [63]. In another study authors observed that MCI patients, who were followed for almost 5 years, showed a mild to moderate increasing trend for the plasma GFAP, thus making this protein a promising biomarker of conversion from MCI to AD [35]. Moreover, increased plasma GFAP was more associated with the clinical incidence of AD (9 to 17 years before diagnosis) than pTau-181 and the NfL (up to 9 years before diagnosis), making this protein the most accurate predictive biomarker for AD from all three [39].

## 3. Parkinson’s Disease

Parkinson’s disease is the second most common (after AD) neurodegenerative disease, with a global prevalence of more than 6 million individuals. This number is consistent with a 2.5-fold increase in prevalence over the past generation, making PD one of the major causes of neurological disability [35].

The key pathological changes in PD include the progressive degeneration of neurons in the substantia nigra pars compacta involved in dopamine transmission. Abnormal deposition of α-synuclein, a major constituent of Lewy bodies and Lewy neurites, also plays a crucial role in the pathogenesis of PD. Lewy bodies are abnormal, insoluble aggregates present inside nerve cells in PD [36]. Additionally, α-synuclein begins aggregating in the olfactory bulb or dorsal nucleus of the vagus nerve then spreads to other brain regions [41]. The first motor symptoms occur when 60–80% of the dopaminergic neurons of the substantia nigra are already lost [64].

Even though the diagnostic criteria mainly rely on clinical symptoms such as bradykinesia, rigidity, and rest tremor, the rate of misdiagnosis is up to 20% due to clinical overlap with parkinsonism or other etiologies. The most characteristic symptoms of PD are tremors, rigidity, and bradykinesia but also loss of smell, constipation, depression, and altered REM phase [65]. Non-motor symptoms of PD often precede motor symptoms, approximately by 5–10 years, and include constipation, REM sleep behavior disorder, and hyposmia [41]. Currently, there is no blood test in daily clinical practice that would predict the risk of the disease or differentiate between different subtypes. However, a growing body of research indicates some proteins that could have potential as blood biomarkers for PD (Table 2).

### 3.1. α-Synuclein Pathology

Notably, α-synuclein seems to be a valuable biomarker for the diagnosis of Parkinson’s disease. It is an abundant neuronal protein expressed mainly in the neocortex, hippocampus, striatum, thalamus, and cerebellum and is known to be especially involved in PD and dementia with Lewy bodies (DLB). It exists in various forms, from unfolded monomers to fibrils. Under normal conditions, α-synuclein binds to a membrane to perform physiological functions or form a tetramer with an α-helical structure that can resist abnormal aggregation [66]. When the balance between α-synuclein generation and clearance is altered, the monomers aggregate to form oligomers. Such oligomers are highly heterogeneous and can produce cytotoxicity through neuroinflammation, mitochondrial dysfunction, endoplasmic reticulum stress, and synaptic impairment. Numerous studies indicate that α-synuclein oligomers with specific conformations may impair neurons and glial cells by damaging organelles and synapses, disrupting protein homeostasis and causing inflammation [66].

Plasma α-synuclein levels differentiated PD patients from healthy controls and from PD patients with lower cognitive scores with high diagnostic accuracy (AUC = 0.6 and 0.63, respectively). Plasma α-synuclein levels were significantly higher in PD patients (15,506.3 ± 8480.8 pg/mL) than in controls (13,057.0 ± 7770.9). Moreover, there was an elevated level of α-synuclein in PD patients with MMSE ≤ 25 compared to the controls. The accumulation of α-synuclein in the periphery is consistent with the “Braak staging” hypothesis, where α-synuclein pathology has been shown to start in the peripheral autonomic nervous system before spreading to the central nervous system [66].

Plasma α-synuclein may also be useful in differentiating different types of PD. According to Ding et al., the plasma level of α-synuclein was significantly higher in PD patients when compared to controls and significantly higher in the postural instability gait difficulty (PIGD) subtype group when compared to the tremor-dominant (TD) subtype group, suggesting that α-synuclein might be more associated with the PIGD subtype and may help in differential diagnosis and proper treatment, as the PIGD subtype is characterized by more severe motor disorders as well as a higher risk of cognitive dysfunction. Moreover, patients with this subtype tend to have less effective responses to dopamine therapy [67].

Although α-synuclein is present in peripheral fluids, it has limited utility due to the fact that the concentration of this protein in blood is influenced by red blood cells [71]. Growing evidence suggests that exosomes can provide more reliable biomarkers for neurodegenerative diseases, including Parkinson’s disease, than other biological fluids since they carry unique, disease-specific cargos reflecting changes characteristic of the disease [72]. It has been previously shown that neuronal α-synuclein exosomes are elevated early in the disease course [73]. In a study performed by Jiang et al., mean serum neuron-derived exosomal α-synuclein was increased by two-fold in prodromal and clinical PD compared to controls, multiple system atrophy, and other neurodegenerative diseases. Exosomal α-synuclein exhibited great performance (AUC = 0.8) in separating clinical PD from controls. Thus, elevated exosomal α-synuclein precedes the diagnosis of PD and aids in differentiation from other neurodegenerative disorders [68]. Another study examined the usefulness of the plasma exosomal α-synuclein/free α-synuclein ratio, which turned out to be significantly higher in PD patients compared to controls. Conversely, there were no differences in plasma total α-syn levels between PD patients and healthy subjects. Moreover, an increase in ratio correlated with disease severity. Thus, the plasma exosomal α-synuclein/free α-synuclein ratio may be useful in the diagnosis and monitoring progression of PD [69].

### 3.2. Other Biomarkers in PD

Similarly to CSF, blood biomarkers typical for AD are also extensively studied in the context of Parkinson’s disease. The same study as mentioned above examined plasma levels of Aβ1-42 in different PD subtypes. It was found that the plasma level of Aβ1-42 was notably lower in PD patients than in controls and significantly lower in the PIGD group than in the TD group. This suggests that a lower level of plasma Aβ1-42 and a higher plasma level of α-synuclein may be used as biomarkers for diagnosis and differentiation of the subtypes of PD. Another study revealed that the p-Tau181/α-synuclein ratio was significantly higher in the PD group compared to controls, with a sensitivity of 97%, specificity of 62%, and AUC 0.9. This proves the potential use of the p-Tau181/α-synuclein ratio as a biomarker in PD [74]. 

AD-related biomarkers are also useful in the prediction of cognitive decline among PD patients. A significant decrease in the Aβ1-42/Aβ1-40 ratio in Parkinson’s disease dementia (PDD) compared to non-demented Parkinson’s disease (PDND) and healthy controls suggests the presence of Aβ pathology in PDD patients. Furthermore, the elevated plasma GFAP and NfL levels preceded altered Aβ1-42/Aβ1-40 and p-Tau181 levels, indicating that plasma GFAP and NfL levels may reflect extensive reactive astrogliosis and neuronal damage in PD before the onset of AD-related neurodegeneration [70,75].

Lin et al., in a follow-up study, investigated whether plasma AD-related biomarkers can predict PD progression. They observed that plasma p-Tau181 levels and the plasma p-Tau181/Aβ1-42 ratio increased with disease duration and that the plasma p-Tau181/Aβ1-42 ratio had a consistently significant correlation with cognitive status in PD. Furthermore, higher baseline plasma p-Tau181 levels predicted faster cognitive decline and motor symptom deterioration in PD patients and in the APOE ε4 carriers but not in non-carriers [70]. There was an inverse association between serum levels of total tau and MoCA score in PD patients. Moreover, serum total tau correlated with CSF total tau, suggesting that the increase of this protein in the blood reflects widespread degenerative processes in PD patients [75].

## 4. Multiple Sclerosis

Multiple sclerosis is an autoimmune, demyelinating, neurodegenerative disorder affecting the central nervous system. Three main types of MS may be distinguished: relapsing remitting (RRMS), primary progressive (PPMS), and secondary progressive MS (SPMS). Usually, RRMS turns into SPMS, which is irreversible due to progressive neurodegeneration. In approximately 85% of patients, the disease starts with the RRMS phase, during which patients experience alternating episodes of neurological dysfunction and recovery, that may last a couple of years with different frequency. Within 25 years, 90% of those patients will convert to SPMS, which is a constant, irreversible neurological decline [76]. The pathologic hallmarks of the disease are demyelination, remyelination, inflammation, neurodegeneration, and the formation of a glial scar. Those features are present in all forms of MS; however, they vary over time [77].

CIS (clinically isolated syndrome) is the first clinical episode with a feature suggestive of multiple sclerosis. It usually presents itself in young adults and affects optic nerves, the brainstem, or the spinal cord. It is suggested that CIS is the first manifestation of MS. Approximately two-thirds of patients with CIS will have episodes of neurological dysfunction and will eventually convert to RRMS [78].

MS usually presents in young adults aged 20–30 years and the most characteristic symptoms are unilateral optic neuritis; partial myelitis; sensory impairments; and brainstem syndromes, such as internuclear ophthalmoplegia, which develop over several days. Diagnosis is made based on a combination of signs and symptoms, radiographic findings (e.g., magnetic resonance imaging T2 lesions), and laboratory findings (e.g., cerebrospinal fluid–specific oligoclonal bands), which are components of the 2017 McDonald Criteria [79]. However, the literature data indicate the growing potential of blood biomarkers instead of CSF, which would help in diagnosis as well as monitoring progression and treatment efficacy (Table 3).

### Biomarkers of Neurodegeneration and Synaptic Dysfunction

Neurofilaments are located in mature, myelinated axons of the white matter but are also present in the grey matter [31]. They are released to the CSF and blood after axonal damage and neurodegeneration in significant amounts [32]. The NfL is the most abundant of the highly conserved neuron-specific structural neurofilament proteins [89] and has been established as a biomarker to assess acute disease activity, monitor therapy response, and predict the course of disability as well as brain and spinal cord atrophy in the treatment of RRMS and PMS [90].

Due to highly sensitive analytic methods, such as SIMOA, it is possible to measure minimal concentrations (pg/mL) and thus enable assays in serum or plasma by specialized laboratories [91]. Multiple studies showed increased levels of the serum NfL in MS, exploring the value of an ultrasensitive single-molecule array. Notably higher sNfL levels were observed in the MS group compared to healthy controls [80]. Similar results were obtained by Cantó et al. In a 2-year follow-up study, plasma NfL levels were higher in MS patients than in healthy controls and were associated with T2 lesion load in MRI examination, as well as a number of gadolinium-enhancing T1 lesions [81]. According to a study conducted by Kjetil Bjornevik et al., levels of the NfL in serum were increased in MS patients compared to healthy controls even 6 years before clinical symptoms of MS occurred, proving that MS has a long prodromal phase and, thus, neuroaxonal degeneration takes place in that phase. Moreover, the clinical onset was associated with a significant increase in sNfL, which may indicate the utility of this biomarker in monitoring disease progression. Those findings show that MS may have a prodromal phase lasting several years and that neuroaxonal damages are already developed at the early stages of the disease [82].

The NfL is also useful in differential diagnosis of MS subtypes and in evaluating the risk of progression from CIS to MS. Serum NfL levels were notably higher in RRMS (8.9 pg/mL) than in CIS patients (4.7 pg/mL) so that it may be considered as a differential parameter. Moreover, higher serum NfL levels increased sensitivity, specificity, and accuracy over gadolinium-enhanced (Gd+) lesions on the brain MRI and OCB to discriminate between MS and CIS [84]. It was observed that the sNfL level was higher in patients with a clinically isolated syndrome or relapsing remitting multiple sclerosis, as well as in patients with secondary or primary progressive multiple sclerosis, than in healthy controls. Moreover, high sNfL concentrations were associated with brain and spinal cord volume loss [83]. It was evaluated that the sNfL has a predictive value in tracking the course of the disease. Manouchehrinia et al. found that an elevated plasma NfL (pNfL) level was associated with increased adjusted rates of EDSS worsening. Moreover, higher NfL levels at the early stages of MS were associated with an increased risk of worsening sustained disability. Finally, increased levels of the NfL were associated with a higher risk of transitioning to progressive MS in relapsing onset patients [85]. These findings suggest that the plasma NfL may provide additional predictive power in the form of an easily accessible biomarker for monitoring disease activity in MS. Compared to healthy controls (10.5 ng/L), serum NfL levels were significantly higher in RMMS patients (16.9 ng/L) and in patients with progressive MS (23 ng/L). Furthermore, patients with relapse or with radiologic activity had significantly higher serum NFL levels than those in remission or those without new lesions on MRI [85]. 

Additionally, the plasma NfL turned out to be useful in monitoring responses to disease-modifying therapies. Kuhle et al. showed that, after treatment with fingolimod, serum NfL levels were notably decreased compared to the placebo group [86]. In a similar study, patients starting fingolimod had reduced plasma NfL levels between baseline and at 12 months and levels remained stable at 24 months, indicating that plasma NfL levels decreased after successful treatment. Thus, the plasma NfL may serve as a biomarker for MS therapy responses [87].

An additional advantage of the NfL as a biomarker is its stability in the measurement. Serum NfL concentrations remained stable after 24 h freezing. Moreover, repeated thawing and re-freezing cycles (up to three times) did not change serum NfL concentration significantly (as measured by SIMOA) [92].

The literature data indicate that, also, GFAP could be a useful biomarker for MS. Blood levels of GFAP are investigated in MS, especially in the context of disease progression, as this protein is a well-established marker of astrogliosis. Serum GFAP levels were increased in PMS compared to RRMS and other non-inflammatory neurological diseases. Moreover, levels of serum GFAP were elevated with increasing MRI-lesion count. Thus, blood GFAP may be a suitable disease progression biomarker [61]. Furthermore, Sharquie et al. showed that patients with active and inactive RRMS showed a higher concentration of serum levels of GFAP than healthy controls. Patients with the RRMS in the active phase also had higher levels of GFAP than those in remission. Thus, GFAP serum levels could serve as a potentially useful biomarker for detecting and monitoring disease stages [88].

## 5. Creutzfeldt–Jakob Disease

Creutzfeldt–Jakob disease (CJD) is the most common prion disease (next to Gerstmann–Sträussler–Scheinker syndrome and fatal familial insomnia), which is a fatal and transmissible neurodegenerative disorder characterized by the misfolding and aggregation of prion protein (PrP). The annual incidence of Creutzfeldt–Jakob disease is one per million. CJD may be inherited (PRNP mutation), acquired by infection, or may occur spontaneously (sporadic Creutzfeldt–Jakob disease; sCJD) [93].

Neuropathologically, it is characterized by spongiform changes in grey matter, with a loss of neuronal cells, astrogliosis, and accumulation of misfolded PrP. The cerebral neocortex is the most severely affected region in CJD pathology and the severity of damage is associated with total disease duration [94]. 

Clinically, CJD is characterized by a rapid progression and patients develop an akinetic mutism state only several months after disease onset. The so-called “classic triad” of symptoms includes rapidly progressive cognitive dysfunction, myoclonus, and periodic sharp-wave complexes on EEG [95]. 

Only neuropathological examination of brain tissue enables definite diagnosis of CJD [96]. Ante-mortem probable or possible CJD is defined based on clinical features, EEG (periodic sharp and slow wave complexes), a positive 14-3-3 assay of CSF, and altered signals on the brain MRI [97]. Accurate and possibly early markers are important in CJD as there is no proven disease-modifying treatment currently available. The use of CSF markers of neuronal damage, especially 14-3-3 and the NfL, as well as real-time quacking conversion assay (RTR-QuIC), significantly increased the diagnostic process [98]. Blood biomarkers have been extensively studied in CJD as they would enable faster diagnosis and differentiation between other dementias (Table 4).

### 5.1. Total Prion Protein

PrP^C^ is the normal form of the protein and is found on the cell membranes. Studies on animal models showed that its cleavage in peripheral nerves leads to activation of myelin repair in Schwann cells so lack of PrP^C^ may cause demyelination. However, the infectious form of PrP (PrP^Sc^) converts other proteins to their infectious form [103].

F. Llorens et al. examined the concentration of total prion protein in the plasma of patients with neurodegenerative dementias. It showed elevated total PrP levels in sporadic CJD (54 ± 25 ng/mL), followed by FTD (46 ± 27 ng/mL), AD (40 ± 29 ng/mL), VaD (37 ± 21 ng/mL), and LBD (33 ± 15 ng/mL), compared to healthy controls (22 ± 10 ng/mL) and the neurological disease control group (28 ± 34 ng/mL). Sporadic CJD cases were discriminated from healthy controls and neurological disease control group patients with high accuracy (AUC 0.92 and 0.85, respectively). Moreover, the authors investigated whether there is an association between total PrP levels and the duration of the disease in sporadic CJD. Mean total PrP concentrations appeared higher at advanced disease stages (48 ± 15 ng/mL at Stage 1, 53 ± 27 ng/mL at Stage 2, 56 ± 25 ng/mL at Stage 3); however, these differences were not statistically relevant [99].

### 5.2. Biomarkers of Neurodegeneration

Experimental data confirmed that the NfL and tau may be applied as diagnostic tools in CJD. A study conducted by Noguchi et al. showed that tau protein levels in serum are markedly higher in the CJD group (193 ± 72.6 pg/mL) compared to AD (0 ± 3.3 pg/mL), non-CJD with rapidly progressive dementia (22 ± 21.8 pg/mL), and the healthy control group (0 ± 9.37 pg/mL). Serum tau may be, therefore, a useful marker to differentiate CJD from AD and non-CJD with rapidly progressive dementia [102].

Among diagnostic groups, the highest plasma NfL and total tau concentrations were detected in CJD (fold changes of 38 and 18, respectively), as compared to healthy controls. Elevated total tau was able to differentiate CJD from all other groups. Both biomarkers discriminated CJD from non-CJD dementias with high diagnostic performance (AUC of 0.93). Thompson et al. conducted a study using the ultrasensitive technique SIMOA to measure serum concentrations of two proteins: tau and NfL. Serum tau concentrations were significantly higher in patients with sporadic CJD compared with healthy controls (median: 6.22 pg/mL vs. 1.56 pg/mL). Serum NfL levels were also markedly increased in sporadic CJD cases (median: 296 pg/mL vs. 14.5 pg/mL). Cut-off values were 2.2 pg/mL for tau and 44.7 pg/mL for NfL, respectively. As for distinguishing sporadic CJD from healthy controls, serum tau yielded a sensitivity of 91% and specificity of 83%, whereas serum NfL had 100% sensitivity and 100% specificity. Thus, NfL separated sporadic CJD cases from healthy controls more precisely. However, an additional advantage of tau was a positive correlation with disease progression [100]. Moreover, positive correlations were observed between the plasma NfL and total tau concentrations, as well as between plasma and CSF concentrations of both biomarkers. In agreement with the previous study, the authors confirmed a significant correlation between plasma total tau, but not plasma NfL, and disease duration [101].

## 6. Conclusions

Neurodegenerative diseases represent a global problem with an increasing incidence rate. Despite many years of research, the exact pathogenesis of them remains unknown. Due to the fact that NDs are still incurable and no effective treatments to even halt or slow down the progression have been developed, there is an urgent need for the earliest possible detection of the disease. The possibility to measure specific proteins in a variety of neurodegenerative disorders allows the identification of the disease even before the onset of the first symptoms. It is especially important in the context of neurodegenerative diseases as treatment is mostly effective only at the earliest stages. Blood biomarkers could be considered reliable measurements to screen for AD. Recent evidence has revealed that particularly plasma phosphorylated tau isoforms and GFAP could be valuable predictors of preclinical AD in cognitively unimpaired amyloid beta+ subjects. Significantly higher plasma pTau-217 and pTau-181 levels preceded changes in CSF biomarkers or amyloid-PET findings. The additional advantage of pTau hyperphosphorylated at different sites (pTau-181, pTau-212, pTau-217, and pTau-231) is the possibility to distinguish AD from patients with other clinically defined neurodegenerative diseases, including FTD, PD, and vascular dementia. Importantly, their levels correlate with CSF measures, thus, after thorough validation, blood pTau assays may eliminate the need for the invasive collection of CSF or costly PET imaging. Biomarkers of neurodegeneration, such as the NfL and GFAP, have been found to be useful in monitoring progression in different types of MS, as well as general biomarkers for neuronal loss and disease progression. Additionally, those biomarkers, especially tau protein, are highly specific for rapidly progressive dementias, such as Creutzfeldt–Jakob disease. In the context of Parkinson’s disease, an interesting tool seems to be blood α-synuclein exosomes due to their high specificity for PD. To conclude, blood-based biomarkers are a major advance in the clinical diagnosis of neurodegenerative diseases and, due to improvements in the technology, they may soon be used for the screening, diagnosing, or monitoring of treatment responses; although, further research is needed to validate the diagnostic utility of those biomarkers and establish standardized criteria for their clinical use [104].

## Figures and Tables

**Table 1:** Blood biomarkers for AD.

Mechanism of Pathology	Biomarker	Direction of Change	Clinical Application	References
Aβ plague deposition	Aβ1-42	↓	Diagnosis:	
			- decreased in AD compared to controls	[22,23,24,25]
			Prognosis:	
			- lower levels in cognitively healthy individuals (18.3 pg/mL), SCD (17.4 pg/mL), and MCI patients (17.6 pg/mL) with a pathological CSF signature	[22]
	Aβ1-42/Aβ1-40 ratio	↓	Diagnosis:	
			- a prescreener for detection of changes in CSF and/or amyloid-PET	[26]
			- detection of cerebral amyloidosis	
			Differentiation:	
			- significantly decreased in MCI and AD patients compared to other dementia types (FTD, VaD, mixed AD/VaD, LBD, PDD, HD, PSP, MSA)	[23]
			Prognosis:	
			- lower levels associated with clinical progression to MCI or dementia among SCD patients	[27]
			- lower levels in cognitively healthy individuals (0.073), SCD (0.070), and MCI patients (0.066) with a pathological CSF signature	[22]
Tau pathology	pTau-181	↑	Diagnosis:	
			- the highest diagnostic accuracy for distinguishing AD dementia from cognitively unimpaired (AUC = 0.91–0.97)	[28,29]
	pTau-212	↑	Differentiation:	
			- significantly higher in the AD MCI and AD dementia group compared to non-AD MCI and SCD groups	[30]
	pTau-217	↑	Diagnosis:	
			- increased in AD compared to controls (AUC 0.98–1)	[31,32]
			Differentiation:	
			- the highest diagnostic accuracy (AUC = 0.93) for distinguishing AD from FTD	[29]
	pTau-231	↑	Prognosis:	
			- notably increased in MCI patients who converted to AD	
			- longitudinal growth correlated with worse cognition and brain atrophy	[33]
			Differentiation:	
			- high diagnostic accuracy for differentiation of AD patients from amyloid-β negative cognitively unimpaired adults (AUC = 0.92–0.94)	[34]
Neurodegeneration	NfL	↑	Diagnosis:	
			- significantly higher in the MCI–AD group and AD compared to non-demented controls	[35,36]
			Prognosis:	
			- higher concentrations associated with accelerated reduction in FDG-PET measures, expansion of ventricular volume, and lower MMSE scores	[37]
Synaptic dysfunction	GFAP	↑	Differentiation:	
			- discriminating Aβ-positive cognitively unimpaired individuals from Aβ-negative cognitively unimpaired individuals with higher accuracy (AUC 0.69–0.86) than CSF (AUC 0.59–0.76)	[38]
			Prognosis:	
			- increased levels associated with clinical incidence of AD (up to 17 years before diagnosis)	[39]
			- increased in cognitively normal older adults with high brain Aβ load (sensitivity 73%, specificity 72%, AUC 0.8)	[38]

CSF, cerebrospinal fluid; AD, Alzheimer’s disease; SCD, subjective cognitive decline; MCI, mild cognitive impairment; FTD, frontotemporal dementia; VaD, vascular dementia; LBD, Lewy body dementia; PPD, Parkinson’s disease dementia; HD, Huntington’s disease; PSP, progressive supranuclear palsy; MSA, multiple system atrophy; FDG-PET, fluorodeoxyglucose-positron emission tomography; MMSE, Mini-Mental State Examination; Aβ, amyloid β; pTau, phosphorylated tau; NfL, neurofilament light chain; GFAP, glial fibrillary acidic protein AUC, area under the curve; ↑, increase; ↓, decrease.

**Table 2:** Blood biomarkers for PD.

Mechanism of Pathology	Biomarker	Direction of Change	Clinical Application	References
α-synuclein aggregation	total α-synuclein	↑	Diagnosis:	
			- significantly higher in PD patients compared to controls	[66,67]
			Differentiation:	
			- higher levels in the PIGD subtype (340.60 ± 56.00 pg/mL) compared to the TD subtype (299.09 ± 65.79 pg/mL)	[67]
	α-synuclein exosomes	↑	Diagnosis:	
			- notably increased in PD compared to controls (cut-off 14.21 pg/mL with 85% sensitivity, 74% specificity, and AUC 0.86)	[68]
			- significantly increased exosomal α-synuclein/free α-synuclein ratio in PD (compared to controls	[69]
			Prognosis:	
			- elevated exosomal α-synuclein/free α-synuclein ratio associated with disease severity	[69]
Aβ plague deposition	Aβ1-42	↓	Differentiation:	
			- significantly lower in the PIGD group than in the TD group	[69]
	Aβ1-42/Aβ1-40 ratio	↓	Differentiation:	
			- decreased in PD patients with dementia compared to PD patients without dementia (sensitivity 71%, specificity 76%, AUC 0.7)	[70]
Tau pathology	pTau-181	↑	Prognosis:	
			- higher levels predict faster cognitive decline and motor symptom deterioration in PD patients	[70]

PD, Parkinson’s disease; PIGD, postural instability gait difficulty subtype; TD, tremor-dominant subtype; AUC, area under the curve; ↑, increase; ↓, decrease.

**Table 3:** Blood biomarkers for MS.

Mechanism of Pathology	Biomarker	Direction of Change	Clinical Application	References
Neurodegeneration and synaptic dysfunction	NfL	↑	Diagnosis:	
			- significantly higher levels in MS patients compared to controls	[80,81,82]
			Differentiation:	
			- higher concentration in RRMS patients compared to CIS	[83,84]
			- higher levels in PMS (median 23 ng/L) than RRMS (median 16.9 ng/L) and controls (median 10.5 ng/L)	[85]
			Prognosis:	
			- elevated levels in MS patients associated with EDSS worsening and higher risk of progression to PMS	[83]
			Treatment efficacy:	
			- notably decreased after treatment with fingolimod	[86,87]
	GFAP	↑	Differentiation:	
			- higher levels in PMS than RRMS	[61]
			- increased in the active RRMS phase (6.47 ± 3.39 ng/mL) compared to patients in remission (5.33 ± 2.82 ng/mL)	[88]

MS; multiple sclerosis; RRMS, relapsing remitting multiple sclerosis; CIS, clinically isolated syndrome; PMS, progressive multiple sclerosis; EDSS, Expanded Disability Status Scale; NfL, neurofilament light chain; GFAP, glial fibrillary acidic protein; ↑, increase.

**Table 4:** Blood biomarkers for CJD.

Mechanism of Pathology	Biomarker	Direction of Change	Clinical Application	References
PrP aggregation	PrP	↑	Differentiation:	
			- the highest PrP level in CJD (54 ± 25 ng/mL) compared to FTD (46 ± 27 ng/mL), AD (40 ± 29 ng/mL), VaD (37 ± 21 ng/mL), and LBD (33 ± 15 ng/mL)	[99]
			- concentration increases as the disease progresses	
Neurodegeneration	Total tau	↑	Diagnosis:	
			- significantly higher CJD patients compared to healthy controls (91% sensitivity, 83% specificity)	[100]
			- eighteen times higher concentration compared to healthy controls (84% sensitivity, 100% specificity)	[101]
			Differentiation:	
			- CJD from AD and non-CJD rapid progressive dementia (viral encephalitis, paraneoplastic syndrome, hydrocephalus, tuberculous meningitis, and multiple cerebral infarction)	[102]
	NfL	↑	Diagnosis:	
			- markedly increased in sporadic CJD compared to controls (100% sensitivity, 100% specificity)	[100]
			- thirty-eight times higher concentration in CJD compared to healthy controls (100% sensitivity, 100% specificity)	[101]

PrP, prion protein; CJD, Creutzfeldt–Jakob disease; AD, Alzheimer’s disease; FTD, frontotemporal dementia; VaD, vascular dementia; LBD, Lewy bodies dementia; NfL, neurofilament light chain; ↑, increase.
